# The Effectiveness in Activating M-Type K^+^ Current Produced by Solifenacin ([(3R)-1-azabicyclo[2.2.2]octan-3-yl] (1S)-1-phenyl-3,4-dihydro-1H-isoquinoline-2-carboxylate): Independent of Its Antimuscarinic Action

**DOI:** 10.3390/ijms222212399

**Published:** 2021-11-17

**Authors:** Hsin-Yen Cho, Tzu-Hsien Chuang, Sheng-Nan Wu

**Affiliations:** 1 Department of Physiology, National Cheng Kung University Medical College, Tainan City 70101, Taiwan; lilyzhou861126@gmail.com (H.-Y.C.); fytg55qq@gmail.com (T.-H.C.); 2 Institute of Basic Medical Sciences, National Cheng Kung University Medical College, Tainan City 70101, Taiwan

**Keywords:** solifenacin (Vesicare^®^), M-type K^+^ current, current kinetics, voltage-dependent hysteresis, M-type K^+^ channel, pituitary cell, hippocampal neuron

## Abstract

Solifenacin (Vesicare^®^, SOL), known to be a member of isoquinolines, is a muscarinic antagonist that has anticholinergic effect, and it has been beneficial in treating urinary incontinence and neurogenic detrusor overactivity. However, the information regarding the effects of SOL on membrane ionic currents is largely uncertain, despite its clinically wide use in patients with those disorders. In this study, the whole-cell current recordings revealed that upon membrane depolarization in pituitary GH_3_ cells, the exposure to SOL concentration-dependently increased the amplitude of M-type K^+^ current (I_K(M)_) with effective EC_50_ value of 0.34 μM. The activation time constant of I_K(M)_ was concurrently shortened in the SOL presence, hence yielding the K_D_ value of 0.55 μM based on minimal reaction scheme. As cells were exposed to SOL, the steady-state activation curve of I_K(M)_ was shifted along the voltage axis to the left with no change in the gating charge of the current. Upon an isosceles-triangular ramp pulse, the hysteretic area of I_K(M)_ was increased by adding SOL. As cells were continually exposed to SOL, further application of acetylcholine (1 μM) failed to modify SOL-stimulated I_K(M)_; however, subsequent addition of thyrotropin releasing hormone (TRH, 1 μM) was able to counteract SOL-induced increase in I_K(M)_ amplitude. In cell-attached single-channel current recordings, bath addition of SOL led to an increase in the activity of M-type K^+^ (K_M_) channels with no change in the single channel conductance; the mean open time of the channel became lengthened. In whole-cell current-clamp recordings, the SOL application reduced the firing of action potentials (APs) in GH_3_ cells; however, either subsequent addition of TRH or linopirdine was able to reverse SOL-mediated decrease in AP firing. In hippocampal mHippoE-14 neurons, the I_K(M)_ was also stimulated by adding SOL. Altogether, findings from this study disclosed for the first time the effectiveness of SOL in interacting with K_M_ channels and hence in stimulating I_K(M)_ in electrically excitable cells, and this noticeable action appears to be independent of its antagonistic activity on the canonical binding to muscarinic receptors expressed in GH_3_ or mHippoE-14 cells.

## 1. Introduction

**Figure 1:**
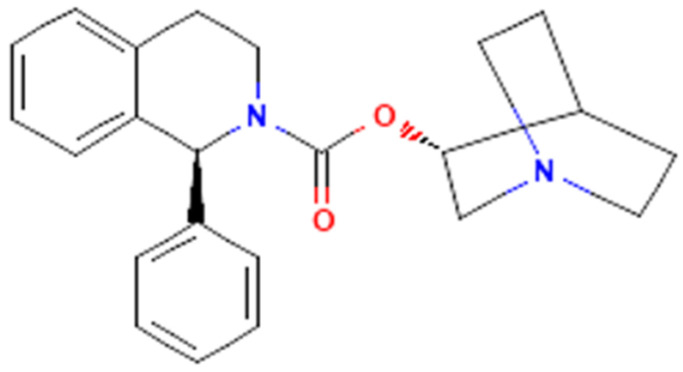
Chemical structure of solifenacin (Vesicare^®^, [(3R)-1-azabicyclo [2.2.2]octan-3-yl] (1S)-1-phenyl-3,4-dihydro-1H-isoquinoline-2-carboxylate, 2(1H)-isoquinolinecarboxylic acid, 3,4-dihydro-1-phenyl-,1-azabicyclo(2.2.2.)oct-3-yl ester, (R-(R*,S*))-905, quinculidin-3′-yl-1-phenyl-1,2,3,4-tetrahydroisoquinoline-2-carboxylate).

Solifenacin (Vesicare^®^, SOL), a member of isoquinolines (Figure 1), has been viewed as an oral anticholinergic (i.e., a competitive muscarinic [M_1_ and M_3_] receptor antagonist) and antispasmodic agent used to treat the symptoms of overactive bladder, neurogenic detrusor overactivity, or urinary incontinence [1,2,3,4,5,6,7,8]. It has been reported to be a muscarinic (M_2_ and M_3_) receptor antagonist that has anticholinergic effects such as relaxation of the detrusor muscle in urinary bladder [9].

Earlier clinical investigations have revealed the efficacy and safety of the antimuscarinic, solifenacin (SOL), for treating patients with overactive bladder or neurogenic detrusor overactivity [1,2,3,4,5,7,8,10,11,12,13,14,15,16,17,18,19]. However, recent evidence has been reported showing that the treatment with SOL could be closely linked to an increased risk of the impairment in cognitive functions [20,21,22,23,24,25,26,27,28,29,30,31]. Therefore, it is pertinent to reappraise the mechanism of SOL actions on electrical behaviors in varying excitable cells, given that its growing clinical use occurs [6,32].

Many types of anterior pituitary cells have been previously demonstrated to secrete acetylcholine [33]. Earlier studies have also revealed that pituitary GH_3_ cells could exhibit the activity of muscarinic receptors and that muscarinic agonists were able to inhibit hormonal secretion through a reduction in intracellular cyclic AMP [33,34,35,36,37,38,39,40]. In these cells, the binding of acetylcholine to M_2_-muscarinic receptor might induce a weak stimulation on the hydrolysis of phosphatidylinositol 4,5-bisphosphate [41]. The binding of acetylcholine to muscarinic receptors in GH_3_ cells was also reported to activate the activity of G protein-coupled K^+^ channels directly [42,43] and to inhibit voltage-gated Ca^2+^ currents [44]. Whether SOL could perturb the electrical activities directly or indirectly through its binding of acetylcholine to muscarinic receptors in pituitary cells is uncertain.

The KCNQ2, KCNQ3, or KCNQ5 gene is viewed to encode the core subunit of K_V_7.2, K_V_7.3, or K_V_7.5 channel, respectively. The enhanced activity of this family of voltage-gated K^+^ channels (KCNQx or K_M_ channels) can generate the macroscopic M-type K^+^ current (I_K(M)_), which is biophysically characterized by current activation in response to low-threshold voltage [45]. Once being activated, this type of K^+^ currents can be sensitive to block by linopirdine and it is demonstrated to exhibit a slowly activating and deactivating property [46,47,48,49,50,51]. Alternatively, targeting I_K(M)_ has been noticeably viewed as an adjunctive regimen for the management of various neurological, smooth muscle, or endocrine disorders closely linked to membrane hyperexcitability, which include cognitive dysfunction, epilepsy, and over-active bladder [47,52,53,54,55]. However, to our knowledge, how and whether this agent can interact directly with K_V_ channels to modify the amplitude and gating of voltage-gated K^+^ currents (e.g., M-type K^+^ current) remain largely unknown.

Therefore, in terms of the considerations stated above, in the current study, we decided to explore the possible perturbations of SOL on I_K(M)_ in pituitary GH_3_ cells and mouse mHippoE-14 hippocampal neurons. Findings from the present observations enable us to reflect that the I_K(M)_ inherent in different cell types could be an additional and yet non-canonical target through which SOL can act to govern the functional activities of the cells involved, presuming that similar in vitro or in vivo findings occur. It thus merit attention that the stimulation of I_K(M)_ and the antagonistic effect on the binding to muscarinic receptors may potentially converge to act on the functional activities of neurons, and neuroendocrine or endocrine cells.

## 2. Results

### 2.1. Effect of SOL on the M-Type K^+^ Current (I_K(M)_) Measured from GH_3_ Cells

**Figure 2:**
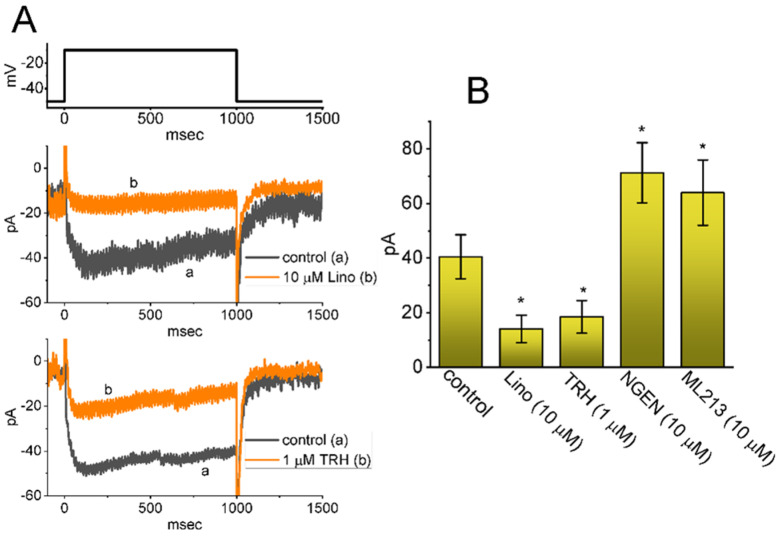
Effect of linopirdine (Lino), thyrotropin releasing hormone (TRH), naringenin (NGEN) or ML213 on M−type K^+^ current (I_K(M)_) recorded from pituitary tumor (GH_3_) cells. These experiments were performed in cells which were kept bathed in high−K^+^, Ca^2+^−free solution containing 1 μM TTX and 0.5 mM CdCl_2_, and we then backfilled the recording electrode by using K^+^−containing (145 mM) solution. (**A**) Representative current traces obtained in the control period (a’s) or during exposure (b’s) to 10 μM Lino (upper part) or 1 μM TRH (lower part). The uppermost part shows the voltage−clamp protocol used. (**B**) Summary bar graph showing effects of Lino, TRH, NGEN, or ML213 on the amplitude of I_K(M)_ in GH_3_ cells (mean ± SEM; *n* = 7 for each bar). Current amplitude was measured at the end of depolarizing pulse from −50 to −10 mV. Statistical analysis was made by ANOVA−1 (*p* < 0.05). * Significantly different from control (*p* < 0.05).

**Figure 3:**
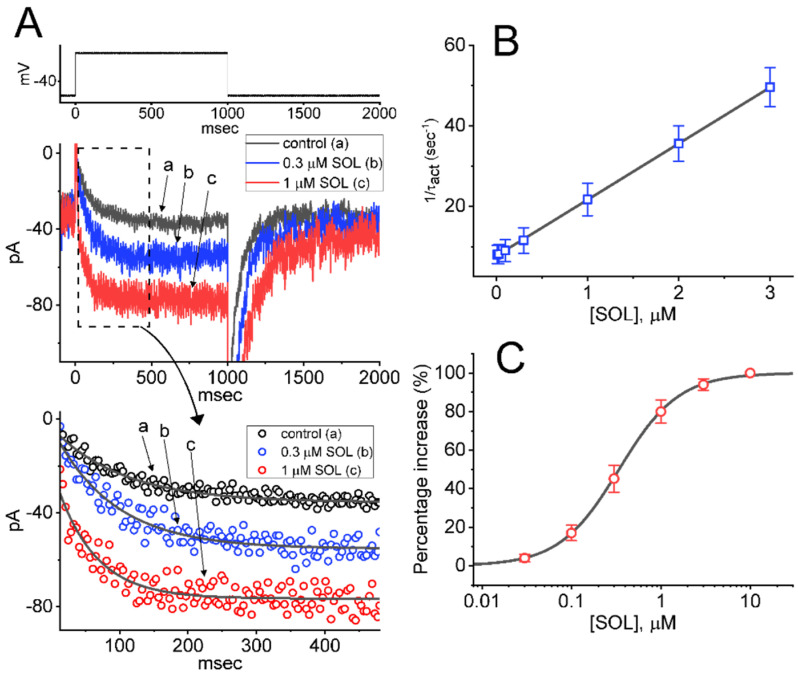
Effect of SOL on I_K(M)_ recorded from GH_3_ cells. This set of experiments was undertaken in cells which was kept bathed in high−K^+^, Ca^2+^−free solution containing 1 μM TTX and 0.5 mM CdCl_2_, and we backfilled the recording electrode by using K^+^−containing (145 mM) solution. (**A**) Representative I_K(M)_ traces obtained in the control period (i.e., SOL was not present; a) and during cell exposure to 0.3 μM SOL (b) or 1 μM SOL (c). The uppermost part denotes the voltage−clamp protocol applied, while the lower part shows the activation time courses of I_K(M)_ taken in the absence (a) and presence of 0.3 μM SOL (b) or 1 μM SOL (c). Current traces in the bottom panel show an expanded record from the dashed box in the top panel, and their trajectories taken from (A) was well fitted by a single exponential (indicated in smooth gray line). Data points (indicated in open circles) with or without the addition of SOL are reduced by 20. (**B**) Kinetic estimate of SOL−stimulated I_K(M)_ identified in GH_3_ cells (mean ± SEM; *n* = 8 for each point). The reciprocal of activation time constant of I_K(M)_ (1/*τ_act_*) derived from exponential fit of the I_K(M)_ trajectory was collated and linearly plotted against the SOL concentration (gray straight line). Forward ($k_{+1}^{*}$) or backward (*k*_−1_) rate constant for the binding scheme, derived from the slope and the *y*−axis of the interpolated line was estimated to be 13.962 sec^−1^μM^−1^ or 7.672 sec^−1^, respectively; thereafter, the K_D_ value (*k*_−1_/$k_{+1}^{*}$ = 0.55 μM) was yielded. (**C**) Concentration−dependent relationship of SOL effect on I_K(M)_ activated by 1−sec long membrane depolarization (mean ± SEM; *n* = 8 for each point). Current amplitude was measured at the end−pulse of each depolarizing step from −50 to −10 mV with a duration of 1 sec. The sigmoidal curve (gray line) indicates the goodness of fit to the Hill equation, as stated in Materials and Methods.

For the first stage of experiments, we intended to determine the possible effect of SOL on the amplitude and kinetics of I_K(M)_ identified in these cells. In attempts to measure the magnitude of I_K(M)_, we kept cells bathed in high-K^+^, Ca^2+^-free solution which contained 1 μM tetrodotoxin (TTX), and the recording pipette was backfilled with a K^+^-containing (145 mM) solution. When the whole-cell configuration was established, we held the examined cell in voltage-clamp mode at the level of −50 mV and a 1-sec depolarizing command voltage to −10 mV was thereafter applied to it. Under these experimental conditions, a specific population of K^+^ currents with a slowly activating and deactivating property was robustly evoked and it has been thus viewed as an I_K(M)_ [48,49,51,56]. This type of I_K(M)_ found in pituitary lactotrophs including GH_3_ cells has been demonstrated to be sensitive to be blocked by thyrotropin releasing hormone (TRH) [48,57]. As demonstrated in Figure 2A,B, the I_K(M)_ in response to step depolarization from −50 to −10 mV was sensitive to inhibition by 10 μM linopirdine (Lino) or 1 μM TRH, while the presence of 10 μM naringenin (NGEN) or 10 μM ML213 increased current amplitude. NGEN or ML213 was previously reported to be an activator of I_K(M)_ [58,59]. Of particular interest, one minute after GH_3_-cell exposure to SOL, the amplitude of I_K(M)_ upon 1-sec membrane depolarization from −50 to −10 mV progressively became increased together with a concurrent decrease in the activation time constant (*τ_act_*) of the current (Figure 3A). For example, the addition of 0.3 or 1 μM SOL increased I_K(M)_ amplitude to 56 ± 7 pA (*n* = 8, *p* < 0.05) or 77 ± 9 pA (*n* = 8, *p* < 0.05), respectively, from control value of 36 ± 6 pA (*n* = 8). Concomitantly, the presence of 0.3 or 1 μM SOL also reduced the *τ_act_* value to 89.1 ± 12.5 msec or 56.7 ± 10.1 msec, respectively, from control value of 123.5 ± 16.8 msec (*n* = 8). After SOL was removed, current amplitude was returned to 39 ± 7 pA (*n* = 7).

Because the I_K(M)_ activation in response to long-last step depolarization tends to be shortened, our next goal was to determine the kinetics of *SOL*-stimulated currents seen in GH_3_ cells. As demonstrated in Figure 3B, as cells were rapidly depolarized from −50 to −10 mV with a duration of 1 sec, it was noticed that exposure to *SOL* resulted in a reduction in the *τ_act_* value in a concentration-dependent manner. This finding can thus be interpreted to reflect that the stimulatory effect of *SOL* on I_K(M)_ seen in GH_3_ cells is explained by the state-dependent activation in situations where the molecule can preferentially bind to the open state (conformation) of the M-type K^+^ (K_M_) channel, on the assumption of the first-order reaction scheme:

(1) $$C\;\begin{array}{c} \alpha \\ ⇄\\ \beta \end{array}\;O\;\begin{array}{c} k_{+1}^{*}\cdot \left[SOL\right]\\ ⇄\\ k_{-1} \end{array}\;O\cdot \left[SOL\right]$$

or

$$\begin{array}{c} \frac{dC}{dt}=\beta \times O-\alpha \times C\\ \frac{dO}{dt}=\alpha \times C+k_{-1}\times O\cdot \left[SOL\right]-O\times \left(\beta +k_{+1}^{*}\cdot \left[SOL\right]\right)\\ \frac{dO\cdot \left[SOL\right]}{dt}=k_{+1}^{*}\cdot \left[SOL\right]\times O-k_{-1}\times O\cdot \left[SOL\right] \end{array}$$

where *α* or *β* represents kinetic constant for the opening or closing of K_M_ channel, respectively; and $k_{+1}^{*}$ or *k*_−1_ is that for forward (on-) or reverse (off-) rate constant of the *SOL* binding, respectively. “*C*”, “*O*”, or “*O* [*SOL*]” denotes the closed, open, or open [*SOL*] state of the channel, respectively. Forward or reverse rate constant ($k_{+1}^{*}$ or *k*_−1_) in this reaction was evaluated from the *τ_act_* values for *SOL*-stimulated modification in the trajectory of I_K(M)_ activation, as described under the Materials and Methods (Figure 3B). The value of $k_{+1}^{*}$ or *k*_−1_ obtained from eight different cells was consequently determined to be 13.962 sec^−1^ μM^−1^ or 7.672 sec^−1^, respectively; thereafter, the value of dissociation constant (K_D_ = *k*_−1_/$k_{+1}^{*}$) was calculated to be 0.55 μM.

The relationship between the *SOL* concentration and the percentage increase of I_K(M)_ was determined and thereafter constructed. In these experiments, each examined cell was held at −50 mV and the depolarizing step from −50 to −10 mV with a duration of 1 sec was delivered to it, and the I_K(M)_ amplitudes during exposure to different concentrations (0.3–10 μM) of *SOL* were measured at the end of depolarizing step. As illustrated in Figure 3C, *SOL* increased I_K(M)_ amplitude in a concentration-dependent fashion. By use of a non-linear least-squares fit to the experimental data, the EC_50_ value required for the stimulatory effect of *SOL* on I_K(M)_ in GH_3_ cells was calculated to be 0.34 μM, a value that was noticeably similar to the K_D_ value estimated above. As such, these emerging data reflect that SOL alone is able to render I_K(M)_ to be sensitive to stimulation attainable in these cells, which appears to be unlinked to its binding to muscarinic receptors.

### 2.2. Comparison in I_K(M)_ Amplitudes Caused by the Presence of SOL, SOL plus Acetylcholine (ACh), SOL plus Iberiotoxin (Iber), SOL plus Apamin (Apa), SOL plus Tolbutamide (TLB), SOL plus Chlorotoxin (ChTx), SOL plus Linopirdine (Lino), or SOL plus Thyrotropin Releasing Hormone (TRH)

**Figure 4:**
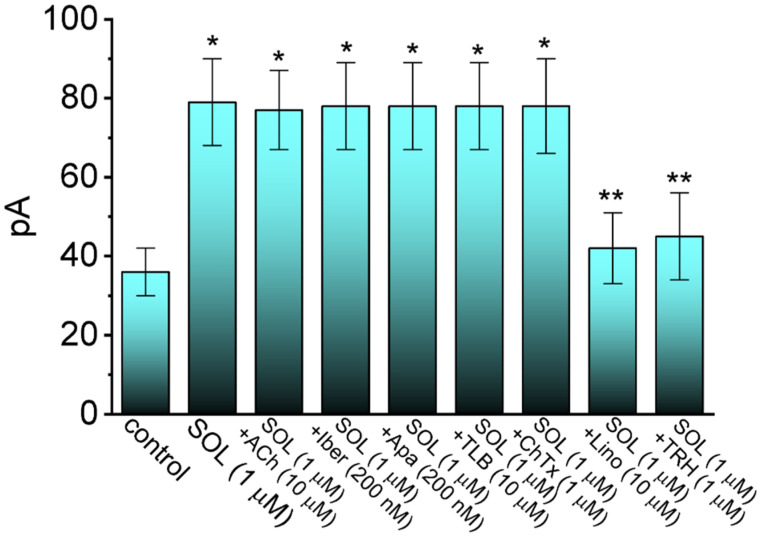
Effect of SOL, SOL plus acetylcholine (ACh), SOL plus iberiotoxin (Iber), SOL plus apamin (Apa), SOL plus tolbutamide (TLB), SOL plus chlorotoxin (ChTx), SOL plus Lino, or SOL plus TRH on the amplitude of I_K(M)_. In these experiments, we bathed GH_3_ cells in high-K^+^, Ca^2+^-free solution and the recording electrode was filled with K^+^-enriched (145 mM) solution. Current amplitude was measured at the end of the depolarizing step from −50 to −10 mV. Each bar represents the mean ± SEM (*n* = 7). * Significantly different from controls (*p* < 0.05) and ** significantly different from SOL (1 μM) alone group (*p* < 0.05).

We continued to examine whether SOL-stimulated I_K(M)_ in GH_3_ cells could be modified by further application of acetylcholine, iberiotoxin, apamin, tolbutamide, chlorotoxin, linopirdine, or thyrotropin releasing hormone. The muscarinic receptor in GH_3_ cells can be activated by acetylcholine [42,43], while iberiotoxin or apamin is an inhibitor of large- or small-conductance Ca^2+^-activated K^+^ channels, respectively, whereas tolbutamide is reported to suppress ATP-sensitive K^+^ channel. Chlorotoxin is known to suppress the activity of Cl^-^ channels, while Lino or TRH is an inhibitor of I_K(M)_ in GH_3_ cells [48,49,50,51]. In the examined cells bathed in high-K^+^, Ca^2+^-free solution, the potential was held at −50 mV and the depolarizing step from −50 to −10 with a duration of 1 sec was applied to the cell. Summary bar graph demonstrated in Figure 4 revealed that cell exposure to 1 μM SOL increased I_K(M)_ amplitude and that neither further addition of acetylcholine (10 μM), iberiotoxin (200 nM), apamin (200 nM), tolbutamide (10 μM), nor chlorotoxin (1 μM) resulted in any adjustments in SOL-stimulated I_K(M)_, while that of Lino or TRH was able to reverse the stimulation of I_K(M)_ caused by SOL. The results indicate that the I_K(M)_ amplitude stimulated by SOL seen in GH_3_ cells is unlinked to its effects on the activity of large- or small-conductance Ca^2+^-activated K^+^ channels or on that of ATP-sensitive K^+^ channels, and that its stimulatory effect on I_K(M)_ is unable to be adjusted by further application of acetylcholine.

### 2.3. Current-Voltage (I-V) Relationship and Steady-State Activation Curve of I_K(M)_ in the Absence and Presence of SOL

**Figure 5:**
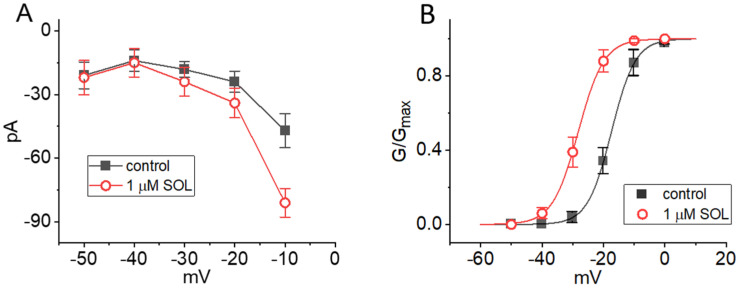
Effect of SOL on the current-voltage (*I-V*) relationship (**A**) and activation curve (**B**) of I_K(M)_ identified in GH_3_ cells. In these experiments, the examined cell was held at −50 mV and the voltage pulses ranging between −50 and 0 mV in 10-mV step were applied to it. (**A**) Averaged *I-V* relationship of I_K(M)_ taken in the absence (■) and presence (○) of 1 μM SOL (mean ± SEM; *n* = 8 for each point). Each data point was taken at the end-point of each voltage pulse. (**B**) Activation curve (i.e., normalized conductance versus membrane voltage) of I_K(M)_ obtained in the control period (■) and during exposure (○) to 1 μM SOL (mean ± SEM; *n* = 8 for each point). The smooth continuous lines give best fit to a modified Boltzmann equation as stated in Materials and Methods. Of note, a leftward shift along the voltage axis in the activation curve of I_K(M)_ recorded from GH_3_ cells is illustrated in the presence of 1 μM SOL, despite no perturbation in the apparent gating charge of the current. The statistical analyses in (**A**) and (**B**) were undertaken by ANOVA-2 for repeated measures, *p* (factor 1, groups among data taken at different level of membrane potentials) < 0.05, *p* (factor 2, groups between the absence and presence of SOL) < 0.05, *p* (interaction) < 0.05, followed by post-hoc Fisher’s least-significance difference test (*p* < 0.05).

We next studied whether the presence of SOL can modify the amplitude of I_K(M)_ measured at different level of membrane potentials. The averaged *I-V* relationship of I_K(M)_ with or without the addition of SOL (1 μM) is illustrated in Figure 5A. The current amplitude was significantly increased as the membrane potential was less negative to −30 mV, and the magnitude of SOL-stimulated current at the level of −10 mV was noted to be greater than that at −20 mV. The relationship of I_K(M)_ conductance versus membrane potential gained in the control period (i.e., SOL was not present) and during cell exposure to SOL (1 μM) was collated (Figure 5B). The smooth sigmoidal curve derived from data sets was reliably fitted with a modified Boltzmann function (described under Materials and Methods). That is, the value of V_1/2_ or q taken in the control period was −17.4 ± 2.1 mV (*n* = 8) or 6.4 ± 0.9 e (*n* = 8), respectively, while that in the presence of 1 μM SOL was −28.2 ± 2.2 mV (*n* = 8) or 6.1 ± 0.9 e (*n* = 8), respectively. The data enable us to reflect that, in addition to increasing I_K(M)_ conductance, the addition of SOL was capable of producing a leftward shift along the voltage axis, albeit with no marked change in the gating charge of the current.

### 2.4. Effect of SOL on Voltage-Dependent Hysteresis (V_hys_) of I_K(M)_ Activated by Long Isosceles-Triangular Ramp Pulse

**Figure 6:**
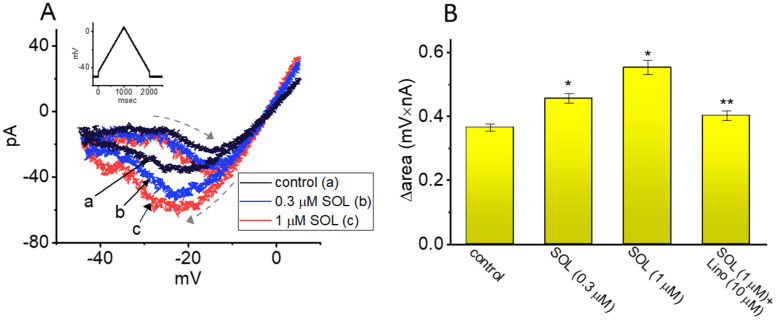
Stimulatory effect of SOL on voltage−dependent hysteresis (V_hys_) of I_K(M)_ in GH_3_ cells. This set of experiments was conducted with an isosceles−triangular ramp pulse. (**A**) Representative current traces activated in response to isosceles−triangular ramp pulse with a duration of 2 sec obtained in the control period (black line, a) and during cell exposure to 0.3 μM SOL (blue line, b) or 1 μM SOL (red line, c). The dashed arrows indicate the distinctive patterns of current trajectory by which time passes as the ramp pulse is applied. The voltage−clamp ramp pulse is illustrated in inset at the left upper corner. (**B**) Hysteretic area (i.e., Δarea) of I_K(M)_ V_hys_ gained in control period (i.e., SOL was not present) or during exposure to SOL and SOL plus linopirdine (Lino). The area encircled by current amplitudes activated in the ascending and descending limb at the voltages between −45 and 0 mV was calculated. Each bar indicates the mean ± SEM (*n* = 7 for each bar). Data analysis was performed by ANOVA−1 (*P* < 0.05). * Significantly different from control (*p* < 0.05) and ** significantly different from SOL (1 μM) alone group (*p* < 0.05).

The V_hys_ of membrane ionic currents (i.e., a lag in the current amplitude as the linear voltage ramp is changed in the opposite direction) has been recently noticed with an impact on the electrical signal events of varying excitable cells [51,60,61,62,63,64,65,66]. In other words, V_hys_ behavior is thought to dynamically adjust the voltage sensitivity and kinetics to optimize channel function for appropriately matching its physiological or pathophysiological role in regulation of AP firing [62,63,65]. Toward this goal, we continued to determine how the presence of SOL might adjust the V_hys_ strength of I_K(M)_. In this separate set of experiments, as soon as the whole-cell configuration was achieved, we maintained the examined cell in voltage clamp at −50 mV, and a long-lasting upright isosceles-triangular ramp pulse with a duration of 2 sec at voltages between −45 and +5 mV (i.e., a ramp slope of ±50 mV/sec) was digitally created and, through DA conversion via Digidata 1440A device, thereafter, delivered to the examined cell at a rate of 0.025 Hz. Of notice, as demonstrated in Figure 6, the I_K(M)_ trajectories elicited in response to the forward upsloping (i.e., voltage change from −45 to +5 mV) ramp pulse and by the backward downsloping (i.e., the change from +5 to −45 mV) as a function of time (as indicated by the dashed arrows in Figure 6A) were markedly distinguishable between these two limbs. In other words, the I_K(M)_ amplitude activated by the upsloping (forward or ascending) limb of the triangular voltage ramp was demonstrated to be smaller than that by the downsloping (backward or descending) end of the ramp. These observations led us to indicate that there was a V_hys_ phenomenon ranging between −45 and −5 mV for this type of recorded currents in GH_3_ cells.

In this study, we continued to quantify the V_hys_ strength of I_K(M)_ on the basis of the area encircled by the curvilinear trajectory in response to the upsloping and downsloping direction in ramp voltage. Figure 6B illustrates a summary of the area under the curve (i.e., Δarea) between the forward and backward currents activated in response to a 2-sec isosceles-triangular ramp pulse. Of notice, when the whole-cell I_K(M)_ was identified, the addition of 0.3 or 1 μM SOL actually increased the area up to 1.2- or 1.5-fold, respectively, while the subsequent application of 10 μM linopirdine, an inhibitor of K_M_ channels, markedly attenuated SOL-induced increase in the area by around 30%. It is conceivable, therefore, that the V_hys_ of I_K(M)_ in these cells can be augmented by the presence of SOL.

### 2.5. Stimulatory Effect of SOL on the Activity of M-Type K^+^ (K_M_) Channels in GH_3_ Cells

**Figure 7:**
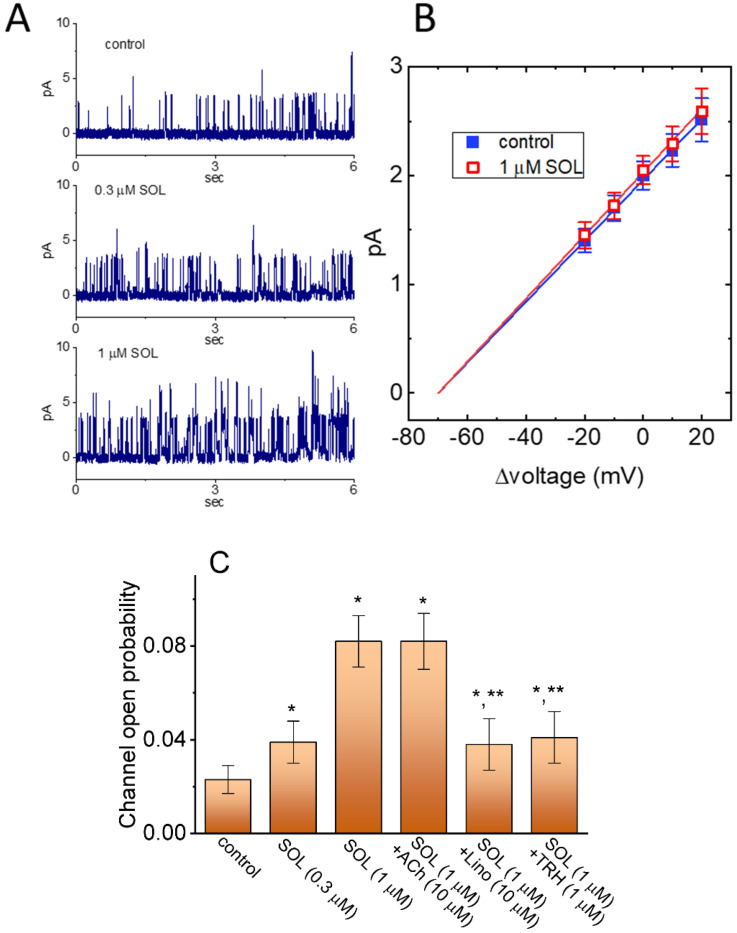
Stimulatory effect of SOL on the activity of M−type K^+^ (K_M_) channels in recorded GH_3_ cells. In this set of cell−attached current recordings, we bathed cells in high−K^+^, Ca^2+^−free solution, while the recording electrode was filled up with low−K^+^ (5.4 mM) solution. (**A**) Representative single K_M_−channel activity obtained in the control period (upper) and during cell exposure to 0.3 μM SOL (middle) or 1 μM SOL (lower). The examined cells were maintained at +20 mV relative to the bath, and the upward deflection indicates the opening event of the channel. (**B**) Averaged *I-V* relationships of single-channel K_M_ currents between the absence (■) and presence (□) of 1 μM SOL (mean ± SEM; *n* = 8 for each point). Notably, no appreciable difference in single−channel conductance of K_M_ channels is depicted in the presence of 1 μM SOL. (**C**) Summary bar graph showing effect of SOL, SOL plus acetylcholine (ACh), SOL plus linopirdine (Lino), or SOL plus thyrotropin releasing hormone (TRH) on the probabilities of K_M_-channel openings (mean ± SEM; *n* = 7 for each bar). Channel activity was measured at the level of +20 mV relative to the bath. Data analysis was performed by ANOVA−1 (*p* < 0.05). * Significantly different from control (*p* < 0.05) and ** significantly different from SOL (1 μM) alone group (*p* < 0.05).

The SOL-induced raise in whole-cell I_K(M)_ stated above could be due to either changes in channel open probability, single-channel amplitude, gating kinetics of the K_M_ channels, or in any combinations. Such reasons thus urged us to assess the single-channel activities of the channels residing in GH_3_ cells. In this stage of cell-attached current recordings, we bathed cells in high-K^+^, Ca^2+^-free solution and the recording electrode used was filled up with low-K^+^ (5.4 mM) solution. As demonstrated in Figure 7, as the examined cell was held at +20 mV relative to the bath, the activity of single-K_M_ channels was robustly detected [51,56,66]. One minute after bath application of SOL, the channel open probability was markedly raised. For example, at the level of +20 mV relative to the bath, the presence of 1 μM SOL significantly increased the probability of channel openings from 0.023 ± 0.006 to 0.082 ± 0.012 (*n* = 7, *p* < 0.01); conversely, no appreciable modification in the single-channel amplitude was shown in its presence (28 ± 2 pS [control] versus 29 ± 2 pS [in the presence of SOL]; *n* = 7, *p* > 0.05). Meanwhile, the mean open time of K_M_ channels in its presence was appreciably increased to 5.2 ± 1.1 msec (*n* = 7, *p* < 0.05) from a control value of 2.8 ± 0.9 msec (*n* = 7). Furthermore, as cells were continually exposed to SOL, subsequent addition of Lino (10 μM) or TRH (1 μM) could attenuate SOL-stimulated channel activity, while that of acetylcholine (10 μM) failed to influence it. However, no detectable change in single-channel conductance of K_M_ channels was observed, although the mean open time of the channel lengthened as well as the channel open probability was elevated.

### 2.6. Effect of SOL on Spontaneous Action Potentials (APs) Recorded from GH_3_ Cells

**Figure 8:**
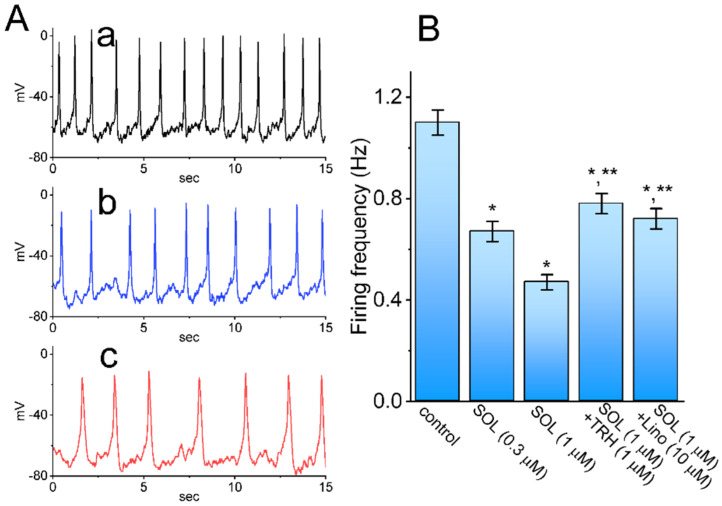
Effect of SOL on spontaneous action potentials (APs) recorded from GH_3_ cells. Whole-cell current-clamp potential recording was carried out in this series of measurements. (**A**) Representative potential traces obtained in the control period (a) and during cell exposure to 0.3 μM SOL (b) or 1 μM SOL (c). (**B**) Summary bar graph showing effect of SOL, SOL plus TRH and SOL plus Lino on firing frequency of APs (mean ± SEM; *n* = 8 for each bar). Data analysis was made by ANOVA-1 (*p* < 0.05). * Significantly different from control (*p* < 0.05) and ** significantly different from SOL (1 μM) alone group (*p* < 0.05).

For another stage of the experiments, the measurements were repurposed to whole-cell potential recordings, in attempts to assess the possible perturbations of SOL on the firing frequency of APs found in these cells. For this stage of measurements, we suspended cells to be bathed in normal Tyrode’s solution containing 1.8 mM CaCl_2_, the recording pipet was filled with K^+^-enriched solution, and whole-cell current-clamp configuration was carried out. As demonstrated in Figure 8, one minute after cell exposure to 0.3 or 1 μM SOL, the firing rate (i.e., spikes/sec) of spontaneous APs was noticeably diminished in combination with concurrent membrane hyperpolarization. For example, the presence of SOL at a concentration of 1 μM overly decreased the firing frequency of spontaneous APs to 0.47 ± 0.03 Hz (*n*= 8, *p* < 0.05) from a control value of 1.10 ± 0.05 Hz (*n* = 8). Moreover, during continued exposure to SOL, subsequent addition of TRH (1 μM) or Lino (10 μM) was able to reverse SOL-mediated inhibition of spontaneous APs effectively. It is likely, therefore, that SOL-mediated decrease in firing frequency of spontaneous APs is mostly mediated through its stimulation of I_K(M)_ identified in GH_3_ cells.

### 2.7. Stimulatory Effect of SOL on I_K(M)_ Present in mHippoE-14 Neurons

**Figure 9:**
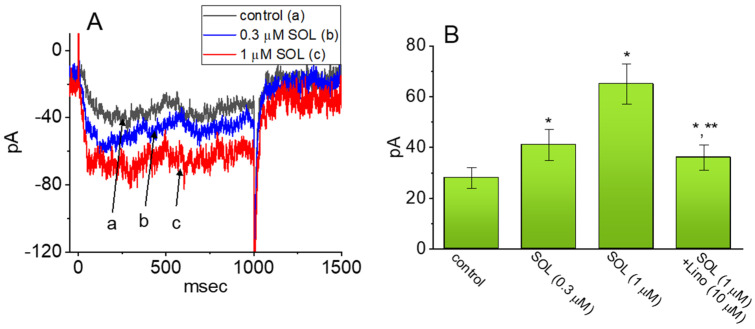
Stimulatory effect of SOL on I_K(M)_ recorded from mouse hippocampal mHippoE-14 neurons. In this set of whole-cell voltage-clamp experiments, cells were bathed in high-K^+^, Ca^2+^-free solution, the recording pipette used was filled up with a K^+^-enriched (145 mM) solution, and the examined cells were depolarized from −50 to −10 mV with a duration of 1 sec. (**A**) Representative current traces obtained in the control period (a) and in the presence of 0.3 μM SOL (b) or 1 μM SOL (c). (**B**) Summary bar graph showing effect of SOL and SOL plus linopirdine (Lino) on the amplitude of I_K(M)_ in mHippoE-14 cells (mean ± SEM; *n* = 7 for each bar). Current amplitude was measured at the end-point of the depolarizing command from −50 to −10 mV. Data analysis was performed by ANOVA-1 (*p* < 0.05). * Significantly different from control (*p* < 0.05) and ** significantly different from SOL (1 μM) alone group (*p* < 0.05). Of notice, the presence of SOL exercises a stimulatory effect on I_K(M)_, and subsequent addition of linopirdine attenuates SOL-mediated stimulation of current amplitude.

Evidence has recently accumulated that the treatment with SOL could be linked to an increased risk of the impairment in cognitive functions [20,21,22,23,24,25,26,27,28,29,30,31]. Earlier reports have also reported the ability of this drug to influence the muscarinic activity in cerebral cortex and hippocampus [67,68]. For these reasons, we further assessed the possible adjustments of SOL on I_K(M)_ in hippocampal mHippoE-14 neurons. This cell line tends to be a homogenous population and it is known to possess the characteristics of embryonic hippocampal neurons valuable for the investigations on different types of neurological disorders [56,69,70,71]. In this series of experiments, we bathed mHippoE-14 cells in high-K^+^, Ca^2+^-free solution which contained 1 μM TTX, and we filled up the recording electrodes by using K^+^-enriched (145 mM) solution. As whole-cell configuration was established, the examined cell was held at −50 mV in voltage-clamp mode and the depolarizing pulse to −10 mV with a duration of 1 sec was delivered to it. As shown in Figure 9 as cells were acutely exposed to different concentrations of SOL, the amplitude of I_K(M)_ activated by such voltage-clamp protocol progressively rose. For example, the presence of 1 μM SOL augmented I_K(M)_ amplitude from 28 ± 4 to 65 ± 8 pA (*n* = 7, *P* < 0.05); and, after removal of SOL, current amplitude was returned to 30 ± 5 pA (*n* = 7). In the continued presence of 1 μM SOL, further application of Lino (μM) attenuated SOL-stimulated I_K(M)_, as demonstrated by an appreciable reduction of I_K(M)_ amplitude to 36 ± 5 pA (*n* = 7, *P* < 0.05). Therefore, it is plausible to assume that indistinguishable from those identified above in GH_3_ cells, I_K(M)_ present in mHippoE-14 neurons, to which I_K(M)_ confers excitability, is subject to stimulation by SOL.

## 3. Discussion

The salient findings noticed in the current investigations are as follows: (a) In pituitary GH_3_ cells, during exposure to SOL, the I_K(M)_ amplitude upon long membrane depolarization was concentration-dependently increased and the activation time course of the current concurrently became shortened; (b) the EC_50_ or K_D_ value of SOL-stimulated I_K(M)_ was calculated to be 0.34 or 0.55 μM, respectively; (c) there is a leftward shift of the steady-state activation curve of I_K(M)_ in its presence; (d) the V_hys_ area of I_K(M)_ activated by isosceles-triangular ramp pulse increased during cell exposure to SOL; (e) the K_M_-channel activity was elevated by adding SOL; however, no change in single-channel conductance of the channel was detected; (f) under current-clamp conditions, the firing frequency of spontaneous APs was measured to be appreciably decreased in the presence of this drug; and (g) the I_K(M)_ inherently in hippocampal mHippoE-14 neurons was also subject to stimulation by SOL. Altogether, regardless of the unresolved detailed ionic mechanism of its actions on K_M_ (or KCNQx) channels, the present results provide an unanticipated and yet non-canonical ionic mechanisms through which the SOL molecule can interact with K_M_ channels to increase whole-cell I_K(M)_ and, consequently, to diminish the firing rate of spontaneous of APs.

One element that is pertinent to notable findings in this study is that the presence of SOL has the propensity to interact with K_M_ channels to increase the magnitude of I_K(M)_ as well as to fasten the activation rate of the current during long depolarizing steps. In other words, although the SOL addition was effective at stimulating I_K(M)_, the activation time course of I_K(M)_ evoked by long-step membrane depolarization became raised. The interaction of SOL with K_M_ channels could also be enhanced by repetitive opening of the channel pore to provide drug access. According to minimal binding scheme, the K_D_ value was yielded to be 0.55 μM, a value which noticeably bears a similarity to effective EC_50_ value needed for SOL-stimulated I_K(M)_. The steady-state activation curve of I_K(M)_ attained in the SOL presence was also found to be shifted along the voltage axis in a leftward direction (i.e., a more negative potential), with no modifications in the gating charge of the current. The mean open time of K_M_ channels was also found to become lengthened in its presence. In this regard, it is plausible to assume that the SOL molecule can preferentially bind to the open state of the K_M_ channel; consequently, the magnitude of I_K(M)_ activated upon long membrane depolarization became elevated during its exposure.

In the present study, the inability of iberiotoxin, apamin, tolbutamide, or chlorotoxin to modify the stimulatory effect on I_K(M)_ caused by the presence of SOL was demonstrated. Iberiotoxin or apamin is viewed to inhibit the activity of large- or small-conductance Ca^2+^-activated K^+^ channels, respectively. Tolbutamide can suppress the activity of ATP-sensitive K^+^ channels, and chlorotoxin is a blocker of Cl^-^ channels. Therefore, it seems unlikely that SOL-mediated stimulation of I_K(M)_ in GH_3_ cells is associated with its perturbations on the activities of large- or small-conductance Ca^2+^-activated K^+^ channels, ATP-sensitive K^+^ channels, or Cl^-^ channels, which were reportedly present in GH_3_ cells. Additionally, in continued presence of SOL, the subsequent addition of acetylcholine failed to reverse SOL-mediated increase in I_K(M)_ amplitude, reflecting that the stimulatory action on I_K(M)_ would not solely be explained by its competitive binding of acetylcholine to muscarinic receptors in these cells, although GH_3_ cells have been previously demonstrated to exhibit the activity of muscarinic receptors [33,34,35,36,37,38,39,40]. Of note, the SOL molecule is structurally similar to tetrahydropyrrolopyrazines demonstrated to activate I_K(M)_ [72], suggesting that 1-phenyl-3,4-dihydro-1H-isoquinoline moiety residing in the molecule is an active site for the binding to the channel.

In accordance with the preceding reports, the V_hys_ phenomenon of I_K(M)_ evoked by the long isosceles-triangular ramp pulse (i.e., the upsloping and downsloping ramp) was revealed in GH_3_ cells [73]. The adjustments of such V_hys_ have been recently noticed to serve a role in fine-tuning the activity of ionic channels (e.g., K_M_ channels) to respond when they are virtually needed [62,63,66,73]. We further determined the possible perturbations of SOL on such dynamic and non-equilibrium properties of I_K(M)_ present in GH_3_ cells. The emerging results allowed us to bespeak that the presence of SOL was able to increase the hysteretic strength of the current efficiently (i.e., Δarea in Figure 6) associated with the voltage-dependent activation of instantaneous I_K(M)_. Under such scenario, it is possible that intrinsic changes in the voltage dependence of the voltage-sensing machinery in K_M_ (KCNQx) channels, namely voltage-sensing domain relaxation would be dynamically modulated during exposure to SOL.

According to previous pharmacokinetic studies, the peak plasma concentrations of SOL with 24.0 ng/mL (0.066 μM) or 40.6 ng/mL (0.11 μM) were reported to reach 3–8 h after long-term oral administration of a 5 or 10 mg SOL dose, respectively [7,74,75,76]. The SOL plasma level was also found to be even higher (i.e., around 52 ng/mL or 0.14 μM) in patients with renal insufficiency [77]; and, it could have a long duration of action as it is usually taken once daily. As such, it is possible that, apart from interfering with the binding to muscarinic receptors, SOL-mediated stimulation of I_K(M)_ is of clinical or therapeutic relevance.

Considering all of the experimental results together, the effects of SOL on I_K(M)_ demonstrated herein appears to be acute and robust in onset; moreover, meanwhile, such stimulatory actions tend to be non-canonical and they are presumably mediated via a mechanism independent of its blockade of muscarinic receptors. These actions probably result in its perturbations on the functional activities of electrically excitable cells (e.g., GH_3_ or mHippoE-14 cells), in the case that in vivo findings occur. Whether the impairment of cognitive function after long-term administration of SOL [20,21,22,23,24,25,26,27,28,29,30,31] could be intimately connected with its stimulation of I_K(M)_ in central neurons remains to be further investigated.

It is worth noting that different types of smooth muscle cells, including smooth myocytes of the urinary bladder, have been demonstrated to be functionally expressed in the activity of K_M_ (KCNQx) channels [52,53,78,79,80,81,82,83,84,85,86,87,88,89]. The SOL-induced interaction with K_M_ channels to modify the magnitude and gating of I_K(M)_ has the propensity to change muscarinic cholinergic activation involved in the micturition reflex, presuming that the in vivo results happen. It turns out that whether the actions of SOL or other structurally similar compounds (e.g., darifenacin) on overactive bladder or neurogenic detrusor over-activity [90] are related to its enhanced actions on K_M_-channel activity [82,83,84,85], warrants further investigations, despite its high-affinity binding to muscarinic receptors.

## 4. Materials and Methods

### 4.1. Chemicals, Drugs and Solutions Used in This Work

Solifenacin (Vesicare^®^, UNII-A8910SQJ1U, YM-905, [(3R)-1-azabicyclo[2.2.2]octan-3-yl] (1S)-1-phenyl-3,4-dihydro-1H-isoquinoline-2-carboxylate, 2(1H)-isoquinolinecarboxylic acid, 3,4-dihydro-1-phenyl-,1-azabicyclo(2.2.2.)oct-3-yl ester, (R-(R*,S*))-905, quinculidin-3′-yl-1-phenyl-1,2,3,4-tetrahydroisoquinoline-2-carboxylate, C_23_H_26_N_2_O_2_, CAS No. 242478-37-1, Solifenacin. Available online: https://pubchem.ncbi.nlm.nih.gov/compound/Solifenacin (accessed on 14 November 2021)) was supplied by MedChemExpress (Asia Bioscience, Taipei, Taiwan), the chemical structure of which is illustrated in Figure 1. Linopirdine (Lino), tetrodotoxin (TTX), thyrotropin releasing hormone (TRH) and tolbutamide (TLB) were acquired from Sigma-Aldrich (Merck, Taipei, Taiwan), and iberiotoxin (Iber) and apamin (Apa) were from Alomone (Asia Bioscience, Taipei, Taiwan). Naringenin (NGEN) was acquired from MP Biomedicals (Cold Spring, New Taipei City, Taiwan), while ML213 (N-(2,4,6-trimethylphenyl)-bicyclo[2.2.1]hepane-2-carboxamide) was from Tocris (Union Biomed, Taipei, Taiwan). Chlorotoxin (ChTx) was kindly provided by Professor Dr. Woei-Jer Chuang (Department of Biochemistry, National Cheng Kung University Medical College, Tainan, Taiwan). Unless stated otherwise, culture media (e.g., Ham’s F-12 or Dulbecco’s modified Eagle’s medium), fetal bovine calf serum, horse serum, L-glutamine, and trypsin/EDTA were supplied by HyClone^TM^ (Thermo Fisher; Level Biotech, Tainan, Taiwan), whereas other chemicals such as CdCl_2_, aspartic acid, and HEPES, were of the best available quality, mostly at analytical grade.

The ion composition of extracellular solution (i.e., HEPES-buffered normal Tyrode’s solution) was as follows (in mM): NaCl 136.5, CaCl_2_ 1.8, KCl 5.4, MgCl_2_ 0.53, glucose 5.5, and HEPES-NaOH buffer 5 (pH 7.4). To record the flowing through I_K(M)_, the patch electrodes were backfilled with the following intracellular solution (in mM): K-aspartate 130, KCl 20, MgCl_2_ 1, KH_2_PO_4_ 1, Na_2_ATP_3_, Na_2_GTP 0.1, EGTA 0.1, and HEPES-KOH buffer 5 (pH 7.2). To measure I_K(M)_, we used a high K^+^-bathing solution containing the following (in mM): KCl 145, MgCl_2_ 0.53, and HEPES-KOH buffer 5 (pH 7.4). To record the activity of single K_M_ channels, the pipette solution was composed of the following (in mM): NaCl 136.5, KCl 5.4, MgCl_2_ 0.53, and HEPES-NaOH buffer 5 (pH 7.4). All solutions used in this work were prepared in deionized water from a Milli-Q^®^ water purification system (Merck Millipore, Taipei, Taiwan). The pipette solution and culture media were always filtered with Acrodisc^®^ syringe filter which contains 0.2-μm Supor^®^ nylon membrane (#4612; Pall Corporation; Genechain Biotechnology, Kaohsiung, Taiwan).

### 4.2. Cell Preparations

The GH_3_ pituitary cell line was supplied by the Bioresources Collection and Research Center (BCRC-60015; Hsinchu, Taiwan), while the embryonic mouse hippocampal cell line (mHippoE-14, CLU198) was from Codarlane CELLutions Biosystems, Inc. (Burlington, ON, Canada) [71]. GH_3_ cell line was originally derived from the American Type Culture Collection (ATCC^®^ [CCL-82.1TM]; Manassas, VA, USA). GH_3_ cells were cultured in Ham’s F-12 medium supplemented with 2.5% fetal calf serum (*v*/*v* percent), 15% horse serum (*v*/*v* percent), and 2 mM L-glutamine, while mHippoE-14 neurons were in Dulbecco’s modified Eagle’s medium supplemented with 10% fetal bovine serum (*v*/*v* percent) and 2 mM L-glutamine. Cells were grown at 37 °C in a humidified environment of 5% CO_2_/95% air.

### 4.3. Electrophysiological Measurements

GH_3_ cells or mHippoE-14 neurons were gingerly harvested and cell suspension was rapidly placed in a customized chamber immediately before the electrical recordings. The recording chamber was positioned on the stage of an inverted DM-IL fluorescence microscope (Leica, Uranus Technology, Taipei, Taiwan) coupled to a digital video system (DCR-TR30; Sony, Tokyo, Japan) with a magnification of up to 1500×. Cells were kept immersed at room temperature (20–25 °C) in normal Tyrode’s solutions containing 1.8 mM CaCl_2_, and the composition of this solution is stated above. The patch-clamp procedure in either whole-cell (voltage- and current-clamp mode) or cell-attached configuration was implemented by using an RK-400 patch amplifier (Biologic, Echirolles, France) [51,91]. When filled with internal solution, patch-clamp glass pipettes had tip resistances ranging between 3 and 5 MΩ and they were made from Kimax-51 capillaries (#34500 [1.5–1.8 mm in outer diameter]; Dogger, Tainan, Taiwan), by using either a PP-830 vertical puller (Narishige, Tokyo, Japan) or a P-97 horizontal puller (Sutter, Novato, CA, USA), and their tips were fire-polished with an MF-83 microforge (Narishige). The potentials were corrected for the liquid–liquid junction potential which emerged when the composition of the pipette solution was different from that in the bath. An anti-vibration air table was used to ensure mechanical stability during the measurements.

### 4.4. Data Recordings

The signals comprising voltage and current tracings were monitored on an HM-507 oscilloscope (Hameg, East Meadow, NY, USA) and stored online in an ASUS ExpertBook laptop computer (P2451F; ASUS, Tainan, Taiwan) at 10 kHz interfaced with a Digidata 1440A converter (Molecular Devices; Bestogen Biotech, New Taipei City, Taiwan), which proceeded for efficient analog-to-digital/digital-to-analog (AD/DA) conversion. During the measurements, the process in data acquisition equipped with this device was controlled by pCLAMP 10.6 program suite (Molecular Devices) run under Windows 7 (Redmond, WA, USA), and the signals were simultaneously displayed on an LCD monitor through USB type-C connection. Current signals were low-pass filtered at 2 kHz with an FL-4 four-pole Bessel Filter (Dagan, Minneapolis, MN, USA) to minimize possible electrical interference. After the recorded data were digitally collected, we off-line collated them using various analytical tools that include LabChart 7.0 program (ADInstruments; KYS Technology, Taipei, Taiwan), OriginPro^®^ 2021 (OriginLab; Scientific Formosa, Kaohsiung, Taiwan) and varying custom-made macros built in Excel^®^ 2021 under Microsoft 365 (Redmond, WA, USA).

### 4.5. Whole-Cell Current Analyses

To evaluate the effect of concentration-dependent stimulation of SOL on I_K(M)_, GH_3_ cells were allowed to be immersed in high-K^+^ (145 mM K^+^), Ca^2+^-free solution. As the whole-cell mode was established, each cell was voltage-clamped at a holding potential of −50 mV, and a 1-sec depolarizing voltage command to −10 mV was delivered to it. The amplitude of I_K(M)_ at the end-pulse of 1-sec depolarizing pulse measured during cell exposure to 10 μM SOL was taken as 100%, and current amplitudes were thereafter compared to those in the presence of different SOL concentrations. The concentration required to increase the I_K(M)_ amplitude by 50% was determined by use of the Hill function:

$$Percentage\;increase\;\left(\%\right)=\frac{E_{max}\times {\left[SOL\right]}^{n_{H}}}{{\mathrm{EC}}_{50}^{n_{H}}+{\left[SOL\right]}^{n_{H}}}$$

where [*SOL*] is the *SOL* concentration applied, *E_max_* the maximal increase in I_K(M)_ caused by *SOL*, EC_50_ the concentration required for 50% stimulation, and *n_H_* the Hill coefficient.

The time-dependent rate constant of forward ($k_{+1}^{*}$) or backward (*k*_−1_) was broadly evaluated from the activation time constant (*τ_act_*) of I_K(M)_ activated by the long depolarizing pulse from −50 to −10 mV. The *τ_act_* values in the presence of different SOL concentrations were approximated by fitting single exponential function to the trajectory of each current trace. Since a Hill coefficient of about 1 was found according to the concentration-dependent curve, the forward or backward rate constant was extended to be determined using the following equation:

$$\frac{1}{\tau_{act}}=k_{-1}+k_{+1}^{*}\;\left[SOL\right]$$

where [*SOL*] is the *SOL* concentration applied, and $k_{+1}^{*}$ or *k*_−1_ was gained from the slope and the *y*-axis intercept at [*SOL*] = 0 of the interpolated regression line, where the relation of the reciprocal time constant of I_K(M)_ activation (1.e., 1/*τ_act_*) versus different *SOL* concentration was constructed.

The relationship of the membrane potential versus the I_K(M)_ conductance gained in the absence or presence of *SOL* was well approximated by a modified Boltzmann function (or the Fermi-Dirac distribution) of the following form:

$$\frac{G}{G_{max}}=\frac{1}{1+exp\left[\frac{-\left(V-V_{1/2}\right)qF}{RT}\right]}$$

where *G* is the I_K(M)_ conductance, *G_max_* the maximal conductance of I_K(M)_, *V*_1/2_ the voltage at which half-maximal activation of the current is achieved, *q* the apparent gating charge, *F* Faraday’s constant, *R* the universal gas constant, and *T* the absolute temperature.

### 4.6. Analyses of Single M-Type K^+^ (K_M_) Channels

Single K_M_-channel currents experimentally measured from GH_3_ cells were collated using pCLAMP 10.7 suite (Clampfit 10.7 subroutine). We determined single-channel amplitude taken with or without the addition of SOL by reliably fitting Gaussian distributions to the amplitude histograms of the closed (resting) or open state. The channel open probabilities were defined as N·PO, which was determined by using the following expression:

$$N\cdot P_{O}=\frac{A_{1}+2A_{2}+3A_{3}+\ldots +{\mathrm{nA}}_{n}}{A_{0}+A_{1}+A_{2}+\ldots A_{n}}$$

where N is a number of active K_M_ channels residing in the patch examined, A_0_ is an area under the curve of an all-points histogram corresponding to the closed (resting) state, and A_1_…A_n_ represents a histogram area that corresponds to the level of distinct open state for 1 to n channels in the patch. The single-channel conductance of K_M_ channels with or without the *SOL* addition was calculated using a linear *I-V* approximation with mean values of single-channel amplitudes measured at the different membrane potentials relative to the bath, while open lifetime distribution of K_M_ channels was fitted with single exponential function.

### 4.7. Curve-Fitting Procedures and Statistical Analyses

Linear (e.g., single-channel conductance) or nonlinear (e.g., Hill or Boltzmann equation and single exponential) curves fitting to experimental data sets demonstrated here was performed from the goodness-of-fit test using either the Solver add-in bundled with Excel^®^ 2021 (Microsoft) or OriginPro^®^ 2021 (OriginLab). The values are provided as means ± standard error of mean (SEM) with sample sizes (*n*), which represents the cell number collected. The Student’s t-test (paired or unpaired) or analysis of variance (ANOVA-1 or ANOVA-2) followed by post-hoc Fisher’s least-significance difference test for multiple-range comparisons, was implemented for the statistical evaluation. Statistical analyses were performed using IBM SPSS version 20.0 (IBM Corp., Armonk, NY, USA). Probability with *p* < 0.05 was considered statistically significant, unless noted otherwise.

## Data Availability

Not applicable.

## Abbreviations

ACh	acetylcholine
ANOVA	analysis of variance
AP	action potential
Apa	apamin
ChTx	chlorotoxin
EC_50_	the concentration required for 50% stimulation
*I-V*	current versus voltage
Iber	iberiotoxin
I_K(M)_	M-type K^+^ current
K_D_	dissociation constant
K_M_ channel	M-type K^+^ channel
Lino	linopirdine
SEM	standard error of mean
SOL	solifenacin (Vesicare^®^)
TLB	tolbutamide
TRH	thyrotropin releasing hormone
*τ_act_*	activation time constant
TTX	tetrodotoxin
V_hys_	voltage-dependent hysteresis
